# Benzydamine hydrochloride: an overview on a well-established drug with news in mechanisms of action

**DOI:** 10.12688/f1000research.144067.1

**Published:** 2024-04-23

**Authors:** Antonio Ferrer-Montiel

**Affiliations:** 1 Instituto de Investigación, Desarrollo e Innovación en Biotecnología Sanitaria de Elche (IDiBE), Miguel Hernandez University, Alicante, 03202, Spain

**Keywords:** Benzydamine hydrochloride, sore throat, oropharyngeal disorders, inflammation, pain relief, nociceptor excitability

## Abstract

Pain and inflammation are the consequences of sore throat, dental and oral procedures, infections, ulcers and head and neck chemotherapy/radiotherapy, and their management is of fundamental importance to avoid distress in patients. Benzydamine hydrochloride (HCl) is a topical indolic nonsteroidal anti-inflammatory drug, endowed with analgesic and anesthetic activity, and with antimicrobial (including both gram-positive and gram-negative bacteria) and antifungal properties (targeting *Candida albicans* and non-albicans strains), used in odontostomatology, otorhinolaryngology, and gynecology for its properties. This molecule has a lipophilic nature, showing high affinity with cell membranes and exhibiting membrane stabilization properties, resulting in local anesthesia, an effect related also to the interaction of the drug with cationic channels. In addition, benzydamine HCl is able to inhibit the production of pro-inflammatory cytokines, with consequent analgesia. Moreover, benzydamine HCl is able to inhibit leukocyte-endothelial interactions and platelet aggregation. Unlike other non-steroidal anti-inflammatory drugs, benzydamine HCl does not inhibit cyclooxygenase or lipoxygenase.

Here we review the most updated clinical data available on benzydamine HCl local application as spray, mouthwash or gargling and evidence of its effectiveness in inflammatory and/or septic conditions in the otorhinolaryngology and odontostomatology settings, with particular reference to sore throat, oral inflammation, dental plaque, tonsillitis/tonsillectomy and chemo- or radiotherapy-induced oral mucositis. Novel formulations for oral administration of benzydamine HCl are also reviewed, including *in situ* gelling formulations to be sprayed onto the damaged oral mucosa. Finally, novel data on the potential role of benzydamine HCl in nociceptor excitability are introduced.

## Introduction

**Figure 1.**
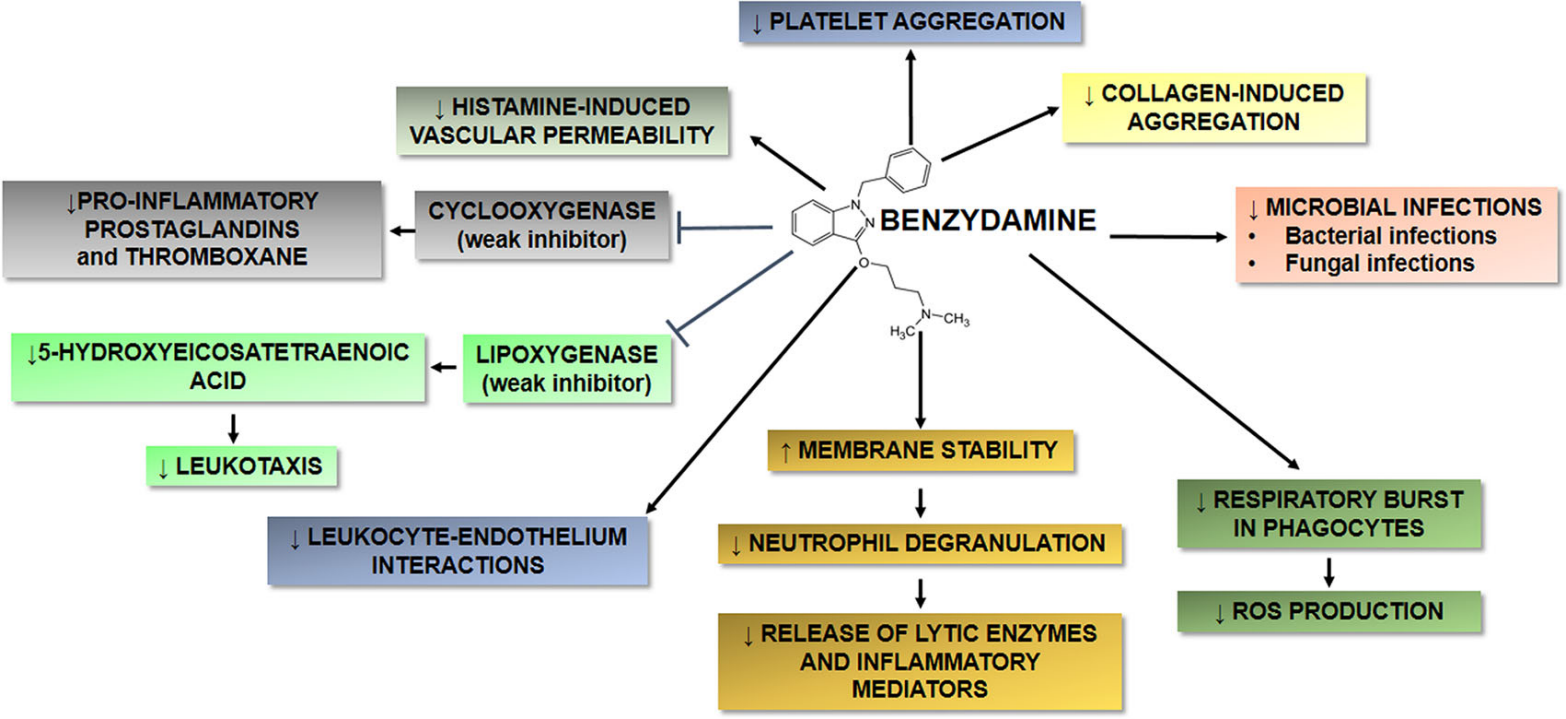
Schematic overview of the main targets and effects of benzydamine HCl on inflammation, microbial infection, membrane stabilization and cell aggregation. Benzydamine HCl shares some properties with other nonsteroidal antiinflammatory drugs, but also displays some unique characteristics. (↓) indicates downregulation; (↑) indicates upregulation.

Benzydamine, available as hydrochloride (HCl) salt, is an indolic nonsteroidal anti-inflammatory drug (NSAID) that shares properties with other NSAIDs, but displays also some peculiar characteristics, including local anaesthetic and analgesic activities, as well as antimicrobial and antifungal properties ( Figure 1). ^1^ Benzydamine HCl is currently formulated as topical preparations for the treatment of oropharyngeal and gynaecological conditions. ^2^ It has been approved also for pediatric use in oropharyngeal conditions. The main characteristics of this molecule, as well as the main available clinical data and perspectives in its use and effectiveness are summarized in this narrative review.

A comprehensive search and critical review have been conducted in PubMed database using the keywords benzydamine hydrochloride, sore throat, oropharyngeal disorders, inflammation, oral mucositis, pain relief through the use of the Boolean operators AND, OR, and identifying the articles relevant to this review. The literature search has been limited to English language articles. Manual search for related articles has been performed as well.

## Pharmacology of benzydamine hydrochloride

### Pharmacokinetics of benzydamine HCl

Benzydamine HCl has high lipid solubility in the unionized form, but low protein-binding capacity, and is consequently taken up by cells with high efficiency. ^1^ Benzydamine HCl is metabolized mainly by conjugation, dealkylation and oxidation, with benzydamine N-oxide as the major metabolite. ^2^

Benzydamine HCl is characterized by a high volume of distribution and a relatively low systemic clearance. ^2^ Plasma drug concentrations of oral benzydamine HCl reach a peak rapidly and decline with a half-life of about 13 h. Less than 20% of the drug results to be bound to plasma proteins. ^3^ The systemic absorption of benzydamine HCl through the skin and non-specialized mucosae is relatively low compared to oral administration, ^2^ thereby limiting unrequired systemic exposure to this drug and the potential for any systemic side-effect.

### The mechanisms of action of benzydamine HCl

Benzydamine HCl shares some properties with other NSAIDs, but exhibits also some distinctive characteristics. Benzydamine HCl has several properties that may contribute to its anti-inflammatory activity, as summarized in Figure 1. Among others, it may exert an anti-inflammatory activity by decreasing the release of pro-inflammatory cytokines, such as tumor necrosis factor-α (TNF-α), interleukin-1β (IL-1β), but not IL-6 and IL-8, without affecting the production of anti-inflammatory molecules, such as IL-10 and IL-1 receptor antagonist (IL-1ra). ^4^ At lower concentrations (3–30 μmol/L), this drug is also able to inhibit leukocyte-endothelial interactions, while at concentration of 10–100 μmol/L, benzydamine HCl may stabilize the mucosal membrane by virtue of its general high affinity for membranes, and may inhibit the release of azurophilic granules from neutrophils, rich in serine proteases, further contributing to its anti-inflammatory effects. ^1^^,^^5^ Benzydamine HCl also reduces the histamine-induced vasodilation and vascular permeability. ^6^ Benzydamine HCl shows antithrombotic activity ^7^ and the inhibition of the collagen-induced aggregation appears to be rather selective *vs.* other NSAIDs. ^4^ Finally, benzydamine HCl is a weak inhibitor of cyclooxygenase and lipoxygenase, and needs high concentrations to inhibit effectively prostaglandin and thromboxane biosynthesis. ^4^

Benzydamine HCl has also anti-microbial properties, supporting its anti-inflammatory activity when applied to the mouth. It proved to be effective in growth inhibition of *Candida albicans* and non-albicans strains, with minimal inhibitory concentrations ranging from 12.5 to 50.0 μg/mL, and with cytotoxic activity at higher concentration, through a membrane-damaging action, consistently with the lipophilic properties of this drug. ^8^ A very recent study confirmed the effectiveness of benzydamine HCl on *Candida albicans* inhibition through the impairment of its adhesion, biofilm formation, regrowth and persistence *in vitro.* ^9^ Benzydamine HCl proved to be effective also against gram-negative and gram-positive bacteria *in vitro* within a few minutes from exposure, at concentrations equal to or lower than those generally used to exert an anti-inflammatory action. ^10^

## The role of benzydamine hydrochloride in prevention and treatment of sore throat: insights from the clinical data

Acute sore throat is a symptom often caused by an inflammation of pharynx, tonsils or nasopharynx and can occur as a part of a common cold of viral origin or can be due to pharyngeal bacterial infections. ^11^ The effectiveness of benzydamine HCl oral rinse for pain relief in acute sore throat has been initially supported by a double-blind study involving 44 patients, where it showed a greater effect on hyperemia and edema at 24 hours *vs.* the placebo solution. ^12^ More recently, a study revealed that chlorhexidine gluconate and benzydamine HCl mouth spray, added to standard antibiotic treatment with penicillin, significantly alleviated the intensity of clinical signs in patients with streptococcal tonsillopharyngitis. ^13^ Of major interest, a novel multicenter, phase IV randomized study proved that both benzydamine HCl 0.3% oromucosal spray and benzydamine HCl 3 mg lozenges were effective in providing a sore throat relief in patients with at least one symptom of upper respiratory tract infection already 2 minutes after a single drug administration, thus addressing the patients’ priority of rapid symptoms’ relief in the setting of upper respiratory tract infections. Clinical efficacy after 7 days of treatment and a good safety profile were also demonstrated. ^14^

Sore throat following tonsillectomy both in adults and children can cause discomfort in the immediate post-operative period. Moreover, since the nerve supplying the tonsil has a branch in the ear, otalgia, i.e. pain along the glossopharyngeal nerve, is commonly referred by patients submitted to tonsillectomy. Benzydamine HCl spray was more effective than placebo in the control of post-operative pain in patients submitted to tonsillectomy in a double-blind trial. ^15^ Another double-blind study confirmed the effectiveness of benzydamine HCl as a local agent in alleviating symptoms of dysphagia and sore throat, but not the earache, thus reducing the requirement of analgesic medication in adult post-tonsillectomy patients. ^16^ However, a prospective, double-blind randomized clinical trial (RCT) did not reveal any difference between mouthwash benzydamine HCl and placebo in intensity and duration of post operative pain, ^17^ suggesting the need for further research studies.

Post-operative sore throat (POST) is an adverse outcome of intubation for general anesthesia that can affect patient’s condition and the care quality, with multifactorial etiology and an incidence ranging from 21% to 66%, in function of the different surgical and anesthetic procedures. ^18^ Different methods have been applied to alleviate POST. Among them, different studies tested the effectiveness of benzydamine HCl by virtue of its analgesic, anesthetic and anti-inflammatory properties. A prospective, double-blind study verified the effectiveness of an approach based on spraying the endotracheal tube cuff and/or the oropharyngeal cavity with benzydamine HCl. The results clearly indicated that spraying benzydamine HCl on the endotracheal tube lowers the incidence and severity of POST without increased benzydamine-related adverse effects. ^18^ These data are consistent with the results of a subsequent meta-analysis of five trials that included 824 patients, showing that the incidence of POST was significantly reduced in the group treated with benzydamine HCl, without major benzydamine-related complications. ^19^ A prospective randomized double-blind comparative study also confirmed the effectiveness of a gargle with benzydamine HCl for 30 seconds before undergoing surgery under general anaesthesia in alleviating the POST, the severity of cough and the voice hoarseness *vs.* a control group treated with placebo. ^20^ Of note, benzydamine HCl gargle proved to be effective in reducing the consumption of propofol, an agent of choice used for sedation during endoscopic retrograde cholangiopancreatography. The combination of propofol and benzydamine HCl mouthwash reduced POST, cumulative propofol consumption/min/kg body weight and desaturation *vs.* the control group that received propofol and water mouthwash. ^21^ These results are of particular interest, as they support the use of a combination of propofol and benzydamine HCl as topical analgesic to reduce propofol consumption and to provide adequate sedation while avoiding propofol side effects, including hypotension and respiratory depression, with a relevant advantage for patients. The combination of sedation with topical anaesthetics in endoscopic procedures is not new and has been tested also in previous studies, where lidocaine in the form of spray or gel was locally applied. ^22^ However, benzydamine HCl mouthwash is becoming an alternative for this approach in the clinical practice as it can induce lower side effects than lidocaine.

Only a few studies did not confirm the effectiveness of prophylactic benzydamine HCl spray on POST following endotracheal intubation. ^23^

## The role of benzydamine hydrochloride in prevention and treatment of chemoradiotherapy-induced oral mucositis: insights from the clinical data

Oropharyngeal mucositis is one of the main adverse events of cancer treatment with chemotherapy or radiation therapy, and is characterized by generalized inflammation, erythema, atrophy and ulceration of oral mucosa. ^24^^,^^25^ Oral mucositis can also follow hematopoietic stem cell transplantation, and can be caused by infections, diabetes and smoking as well. Oropharyngeal mucositis can induce several debilitating symptoms and functional impairment, with consequences on body weight and nutritional status. Moreover, cancer patients could need an unscheduled temporary stop of treatments due to severe oral mucositis, with the risk of a negative impact both on treatment outcome and on survival rates. Oral care is of great importance for prevention or limitation of oropharyngeal mucositis, as it can have an interplay with microbiota and oral infections, including candidiasis and herpes simplex virus-1 infections. ^25^ Benzydamine HCl is widely used in the control of oral mucositis, thanks to its capacity to interact with different inflammation pathways and at the same time to act on pain. The Clinical Practice Guidelines for mucositis issued by the Multinational Association of Supportive Care in Cancer and by the International Society of Oral Oncology recommend the use of benzydamine HCl mouthwash for the prevention of oral mucositis in head and neck cancer (HNC) patients receiving radiotherapy, without concomitant chemotherapy. A recent systematic review of 11 papers further supported the use of benzydamine HCl mouthwash in the prevention and/or treatment of oral mucositis in patients submitted to radiotherapy while also adding chemotherapy. ^26^^,^^27^ However, another systematic review of interventions used to mitigate the radiotherapy-induced oral mucositis in HNC patients did not support the use of benzydamine HCl or other interventions, such as honey and oral glutamine, in radiation-induced oral mucositis. This discrepancy highlights the need for additional high-quality studies with a consensus on the methodology to reduce heterogeneity, and an examination of the cost effectiveness of the interventions. ^28^ Indeed, the discrepancies among study results may be due to dosing and timing of product administration, type of cancer and of patients characteristics, as well as to heterogeneity in anticancer treatments. Several other clinical studies supported the positive effects of benzydamine HCl in preventing and/or reducing the severity of oral mucositis in cancer submitted to radiotherapy. Among others, Chitapanarux *et al*. demonstrated the superiority of benzydamine HCl *vs.* sodium bicarbonate in reducing the severity of oral mucositis in locally advanced HNC patients treated with high-dose radiotherapy concurrently with platinum-based chemotherapy. ^29^ Similar positive results have been obtained by Rastogi *et al.* in the prevention of oral mucositis in HNC patients treated with radiotherapy (>50 Gy) without chemotherapy, while the efficacy of benzydamine HCl in patients receiving concurrent chemotherapy was not confirmed. ^30^ Positive results have been obtained also in pediatric patients affected by chemotherapy-induced oropharyngeal mucositis. ^31^

This overview mostly supports the beneficial effect of benzydamine HCl in oral mucositis following anticancer treatments. Importantly, to the best of our knowledge, there is no evidence of any interference of benzydamine HCl with the activity of anticancer treatments. Moreover, the treatment with benzydamine HCl has been proved not only effective, but also safe and well tolerated in the setting of radiation-induced oral mucositis. ^32^

## The role of benzydamine hydrochloride in prevention and treatment of oral disorders: insights from the clinical data

### Aphthous stomatitis

Recurrent aphthous stomatitis (RAS) is a common and painful disease of oral mucosa of unknown etiology and pathogenesis, not associated with systemic diseases and characterized by the formation of round or oval mouth ulcers.

Since the underlying cause triggering RAS remains to be elucidated, at present the emergence of new lesions cannot be prevented, and therapy focuses mainly on pain reduction, through corticosteroids and topical analgesics plus antiseptics. ^33^ A trial involving patients with minor RAS and comparing the effectiveness of 0.15% benzydamine HCl, 0.2% aqueous chlorhexidine and placebo mouthwashes administered consecutively for 3 months/each, for a total of 9 months, did not reveal any difference between the treatments tested. However, a subgroup of patients preferred the benzydamine HCl preparation because of the temporary topical anesthetic effect, which gave some pain relief. ^34^ A greater control of pain by benzydamine HCl *vs.* other proprietary agents was also highlighted in a study involving hospital out-patients with aphthae in the UK. ^35^ More recently, a prospective, parallel, double-blind, active control, preliminary study compared the flavonoid quercetin with 0.15% benzydamine HCl (Tantum oral rinse) in patients with RAS. The study revealed that both the drugs led to a similar reduction in pain scores from baseline up to day 7 during the treatment period, despite quercetin was able to significantly accelerate ulcer healing *vs.* benzydamine HCl. ^36^

### Dental plaque

Dental plaque is a microbial biofilm adhering to the tooth surfaces and composed by microorganisms and by an extracellular matrix, *i.e.* a mix of molecules derived from both the biofilm microorganisms and from the host. ^37^ Dental plaque is the most important etiological factor for oral diseases and is known to have also systemic effects on health. Oral antiseptics in mouth-rinse formulations are often used to reduce the microbial load in the oral cavity and can play an important role in preservation of oral health, together with mechanical oral hygiene procedures. ^38^ Benzydamine HCl 0.15% as a mouthwash was well tolerated with no reported side effects in a double-blind RCT, and was as effective as chlorhexidine digluconate 0.2% in reducing gingival inflammation due to plaque accumulation, thus controlling further disease progression. ^39^ However, these data were not confirmed by another study comparing chlorhexidine and benzydamine HCl administered as oral sprays to patients affected by gingival inflammation. ^40^

Oral antiseptics can potentially cause irritation and have cytotoxic effects on cells of oral mucosa. The cytotoxic effect of benzydamine HCl has been recently assessed on primary human gingival fibroblasts in parallel with other oral antiseptics. The 2,3-bis (2-methoxy-4-nitro-5-sulfophenyl)-5-[(phenylamino) carbonyl]-2H-tetrazo-lium hydroxide (XTT) cytotoxicity test revealed low cytotoxic effects of benzydamine HCl on fibroblasts, suggesting that it can be used safely to achieve faster wound healing for conditions such as post-operative period. ^41^

### Periodontal surgery

Periodontal surgery aims to prevent or improve different types of defects in the bone, gingiva or alveolar mucosa. Acute pain is one of the most frequent postoperative complications after periodontal surgery, and several analgesics have been tested so far. In this setting, a recent RCT compared the efficacy of 0.15% benzydamine HCl mouthwash *vs.* 400 mg ibruprofen tablets in the reduction of postoperative pain in a period of 24 hours following the crown lengthening, a surgical procedure that addresses excessive gingival display. The trial revealed comparable results for the level of pain in the two groups of patients treated with either benzydamine HCl or ibruprofen; however the investigators supported the use of benzydamine HCl mouthwash to decrease pain after periodontal surgery, because of its fewer side effects and lower price *vs.* ibuprofen. ^42^ Another double-blind RCT involving patients affected by periodontitis demonstrated the superiority of 0.15% benzydamine HCl *vs.* 50 mg diclofenac sodium tablets in reduction of pain perceived by patients submitted to periodontal surgery. ^43^

## The potential role of benzydamine hydrochloride in nociceptor excitability: insights from novel preclinical data

In addition to the anti-inflammatory, analgesic and antimicrobial effects described above ( Figure 1), some studies are revealing the ability of benzydamine HCl to block the neuronal excitability as well. A first indication came from a study investigating the effect of four different NSAIDs, including benzydamine HCl, on neuronally evoked contractile responses to a submaximal dose of prostaglandin E1 (PGE1) or cholecystokinin-octapeptide (CCK-8) in isolated guinea-pig ileum. All the four NSAIDs tested in the study were able to inhibit the CCK-8- or PGE1-induced contractile response in the isolated guinea-pig ileum at very low concentration, thus indicating their ability to interfere with a neuronally-evoked response. Moreover, all the NSAIDs tested were able to depress the opioid system activated by PGE1 or CCK-8. ^44^ A more recent study verified the effect of benzydamine HCl on nociceptor excitability in primary cultures of sensitized rat nociceptors treated with the anti-mycotic compound econazole. This drug, belonging to the imidazole class, is able, at a clinically relevant concentration, to modulate directly the cytosolic Ca ^2+^ homeostasis. Overall, the consequence of econazole action on sensory neurons is an increase in their excitability, augmenting the triggering of action potentials in silent nociceptors and, more evidently, in ATP-sensitized nociceptors, consistently with the higher Ca ^2+^ fluxes. Of interest, a treatment with benzydamine HCl *in vitro* was able to inhibit the econazole-induced nociceptor excitability through the blockade of voltage-gated Na ^+^ (Na _v_) channels. ^45^ These data suggest an alternative pathway to inflammatory cytokine inhibition for the analgesic activity of benzydamine HCl.

Additional studies tested the capacity of benzydamine HCl to decrease the neuronal activity in an inflammatory sensitized neuronal model through electrophysiological recordings. Nociceptors were sensitized through a 24-hours incubation with an inflammatory soup containing histamine, serotonin, ATP and Prostaglandin E2. Action potential firing was induced by acute exposure to an identical inflammatory soup and acid pH, in order to mimic a local inflammatory process. Benzydamine HCl showed a dose-related inhibition of neuronal excitability mediated by either the inflammatory cocktail or the acidic pH in sensitized nociceptors and in basal conditions. Of note, higher potency was shown under inflammatory sensitized conditions compared to basal conditions. ^46^ These novel results support the idea of complementary and synergistic effects of benzydamine HCl in the treatment of local inflammatory symptoms as well as in the painful processes, not only by reducing the inflammation cascade but also by reducing the inflammatory-mediated neuronal signaling.

## The safety of benzydamine HCl administration as spray, mouthwash or gargling: insights from the clinical studies

The safety of benzydamine HCl when administered as spray, mouthwash or gargling in the setting of otorhinolaryngology and odontostomatology, has been verified by several studies. Among them, a multicenter phase IV study showed that both benzydamine HCl 0.3% spray and benzydamine HCl 3 mg lozenges formulations were well tolerated and showed a good safety profile, without any clinically significant abnormality when administered to patients with acute sore throat. ^14^ The absence of significant adverse events related to benzydamine HCl has been showed also by a systematic review and meta-analysis of 13 RCT assessing the efficacy and safety of topical application of benzydamine HCl to prevent POST. ^47^ The safety of benzydamine HCl oral spray administered as adjuvant therapy was further confirmed in a study including 210 children and adults submitted to tonsillectomy, adenoidectomy, or both, when compared to a similar group receiving a *Salvia officinalis* preparation. ^48^ Finally, benzydamine HCl oral spray showed proper safety and was well tolerated, with no serious adverse events reported, when administered to patients with an acute tonsillopharyngitis associated with a common cold. ^49^ Generally, the most commonly reported adverse effects after a topical administration of benzydamine HCl were mild numbness in tissues and tingling sensation in oral cavity after the rinse. These effects are presumably related to the mechanism of action of benzydamine HCl, characterized by a marked, local anesthetic activity, with a rapid onset and transience. ^50^^,^^51^

## Research insights for novel benzydamine HCl formulations

Some studies are testing novel formulations for the administration of benzydamine HCl. An *in-situ* gelling formulation of benzydamine HCl, whose composition includes a thermosensitive polymer to be sprayed onto the damaged oral mucosa by self-administration, showed a sustained drug release and reduced the number of daily administrations of benzydamine HCl, while protecting the damaged mucosa from mechanical and chemical solicitations. ^52^ The chemical and physical characteristics of benzydamine HCl also make this drug ideal for nanosponge complexes, *i.e.* stable nanostructures able to cross multiple barriers, as the pore size of the oral cavity is 2 μm, and particles that are less than this pore size can cross the mucus membrane. ^53^ A recent study tested in the oral mucosa of murine models the local administration of benzydamine HCl in nanosponge-loaded hydrogels, able to increase the drug stability and to make its release more target-specific, for a potential application to mouth ulcers. ^54^ A mucoadhesive buccal film for the mucosal delivery of benzydamine HCl has been recently developed for the treatment of aphthous stomatitis. The film showed adequate physicochemical properties and was able to provide prolonged residence time and sustained delivery *vs.* conventional therapies. ^40^ Finally, nanoparticles composed by chitosan, a linear polysaccharide, have been recently developed and tested *in vitro* for benzydamine HCl delivery. ^55^

An easier application and a higher contact time of benzydamine HCl with the oral mucosa thanks to the novel formulations described above could lead to a greater local bioavailability of the drug with a consequent grater control of pain and inflammation and/or infection, greater management of drug administration and a decrease of potential side effects.

## Conclusions

Pain and inflammation are the consequences of sore throat, dental and oral procedures, infections, ulcers and HNC chemotherapy/radiotherapy, and their management is of fundamental importance to avoid distress in patients. The pharmacokinetics and clinical data summarized in this review revealed that the topical application of benzydamine HCl has several advantages, as the drug is well tolerated and effective, with a marked reduction of the side effects and complications potentially occurring during the systemic administration of other NSAIDs. Benzydamine HCl proved to be safe and efficient in pain relief, and novel properties and formulations are currently under investigation with promising results and new interesting perspectives for this well-established drug.

## Ethics and consent

Ethical approval and written consent were not required.

## Data Availability

No data are associated with this article.
