# Supplementary material for: Benzydamine hydrochloride: an overview on a well-established drug with news in mechanisms of action
Source: F1000Res. 2025 May 22;13:350. Originally published 2024 Apr 23. [Version 2] doi: 10.12688/f1000research.144067.2 (PMC12203043; doi:10.12688/f1000research.144067.2)
Supplement: Peer review history

# Reviewer report: Reviewer response for version 2

**DOI:** 10.5256/f1000research.182442.r393573

**Published:** 2025-06-26

**Authors:** Saai Ram Thejas

**Affiliations:** 1 RVM Institute of Medical Sciences, Telangana, India

**SUMMARY**

 The article is well written and encompasses a whole lot of points about various uses of Benzydamine.

 Usage in oral cavity inflammation/infection, mucositis, surgery, POST has been explained.

 Various ways of administration have also been explained. 

 It has been compared to chlorhexidine and lidocaine and the pros and cons have been explained too.

 What could be added here is the use of Benzydamine in Post Operative Nausea and Vomiting (PONV).

 Otherwise, excellent article.

Is the review written in accessible language?

Yes

Are all factual statements correct and adequately supported by citations?

Yes

Are the conclusions drawn appropriate in the context of the current research literature?

Yes

Is the topic of the review discussed comprehensively in the context of the current literature?

Yes

Reviewer Expertise:

Rhinology, Laryngology, Otology, Newer advances in management of medical conditions

I confirm that I have read this submission and believe that I have an appropriate level of expertise to confirm that it is of an acceptable scientific standard.

# Reviewer report: Reviewer response for version 1

**DOI:** 10.5256/f1000research.157806.r368800

**Published:** 2025-03-27

**Authors:** Muhammet Davut Arpa

**Affiliations:** 1 Istanbul Medipol University, Istanbul, Turkey

This review provides a comprehensive overview of the pharmacological properties, mechanisms of action, clinical efficacy, and novel formulations of benzydamine hydrochloride (HCl). The effects of topical use are discussed in detail, particularly in conditions such as oral and throat inflammations and chemotherapy-induced oral mucositis. The article is generally well-structured, clearly written, and supported by relevant literature. However, there are several points I would like to highlight:

- Oral administration is typically associated with gastrointestinal (GI) delivery of a drug. However, the current review focuses on both oral and oromucosal/buccal/intraoral routes of administration. Therefore, I suggest reviewing the terminology used to ensure consistency and clarity in this context.
- Including more recent publications (post-2020) is essential to enhance the timeliness and relevance of the review.
- Although clinical trials are described in the text, they are not presented in a tabular format. Summarizing key details such as effect size, sample size, and statistical significance in a table would significantly improve readability and provide added value for the reader.
- Most of the clinical data emphasize positive outcomes, while negative or inconclusive results (e.g., studies showing a lack of efficacy) are rarely discussed. It would be beneficial to include a discussion on studies where the treatment was ineffective and possible reasons behind those outcomes.
- While novel formulations such as nanoparticles, gels, or films are briefly mentioned, experimental or clinical data supporting these formulations are limited. The review would be strengthened by a more detailed discussion of the advantages and limitations of these newer delivery systems. Additionally, recent formulation studies should be incorporated to reflect current research trends. Examples include (refer 1to 3).

Is the review written in accessible language?

Yes

Are all factual statements correct and adequately supported by citations?

Partly

Are the conclusions drawn appropriate in the context of the current research literature?

Yes

Is the topic of the review discussed comprehensively in the context of the current literature?

Partly

Reviewer Expertise:

Pharmaceutical technology, oromucosal administration

I confirm that I have read this submission and believe that I have an appropriate level of expertise to confirm that it is of an acceptable scientific standard, however I have significant reservations, as outlined above.

## References

[1] : Novel benzydamine hydrochloride and chlorhexidine gluconate loaded bioadhesive films for local treatment of buccal infections. *Journal of Drug Delivery Science and Technology* .2023;84: 10.1016/j.jddst.2023.104497 10.1016/j.jddst.2023.104497

[2] : Preparation and In vitro, Ex vivo Evaluation of Benzidamine Hydrochloride Loaded Fast Dissolving Oral Strip Formulations: Treatment of Oral Mucositis Due to Side Effects of Chemotherapy and Radiotherapy. *Letters in Drug Design & Discovery* .2023;20(8) : 10.2174/1570180820666230207122755 1147-1157 10.2174/1570180820666230207122755

[3] : Spray-Dried Chitosan Hydrogel Particles as a Potential Delivery System for Benzydamine Hydrochloride. *Gels* .2024;10(3) : 10.3390/gels10030189 10.3390/gels10030189 38534607 PMC10970358

# Reviewer report: Reviewer response for version 1

**DOI:** 10.5256/f1000research.157806.r279009

**Published:** 2024-06-05

**Authors:** Jian-Xin Liu

**Affiliations:** 1 College of Pharmacy, Hunan University of Medicine, Huaihua,, China

This article provides an overview of benzydamine hydrochloride and a detailed analysis of its mechanism of action. Benzamine hydrochloride has been proven to be safe and effective in anti-inflammatory, antibacterial, and pain relief, and its research is an interesting topic. However, the article still has the following issues to consider.

******Comment****** ******1****** ******“****** ******Unlike****** ******other non-steroidal anti inflammatory drugs, benzydamine HCl does not inhibit cyclooxygenase or lipoxygenase.****** ******”******

 The ending with " Unlike other non-steroidal antiinflammatory drugs, benzydamine HCl does not inhibit cyclooxygenase or lipoxygenase." in the abstract is too abrupt, and a sentence can be added at the end. Unlike other nonsteroidal anti-inflammatory drugs, what happens if cyclooxygenase is not inhibited, and whether it improves or deteriorates.

******Comment****** ******2****** ******“****** ******The mechanisms of action of benzydamine HCl****** ******”******

 The description of the mechanism of action of benzydamine HCl in "The mechanisms of action of benzydamine HCl" is too simplistic.

******Comment****** ******3****** ******“The role of benzydamine hydrochloride in prevention and treatment of sore throat: insights from the clinical data****** ******”******

 It is best to add a table to make it clearer and more clear. How does benzidine hydrochloride affect these diseases of throat pain, and what data supports this viewpoint, presented in a table format for a more intuitive understanding.

******Comment****** ******4 ****** As a review paper, the literature cited in this paper is too outdated to reflect the latest research progress. Is there any literature support from the past three years.

******Comment****** ******5****** ******“Only a few studies did not confirm the effectiveness of prophylactic benzydamine HCl spray on POST following endotracheal intubation.”******

 Is there any other literature here that proves this drug is ineffective?

******Comment****** ******6****** ******"The role of benzydamine hydrochloride in prevention and treatment of sore throat****** ******”******

 There is a lack of more in-depth insights into the clinical data here, and it is recommended to delete 1~2 articles inserted into the literature.

******Comment****** ******7****** ******“****** ******Research insights for novel benzydamine HCl formulations****** ******”******

 There is little description of the new dosage form here, and there is a lack of recent advances.

 Abstract

 Pain and inflammation are the consequences of sore throat, dental and oral procedures, infections, ulcers and head and neck chemotherapy/radiotherapy, and their management is of fundamental importance to avoid distress in patients. Benzydamine hydrochloride (HCl) is a topical indolic nonsteroidal anti-inflammatory drug, endowed with analgesic and anesthetic activity, and with antimicrobial (including both gram-positive and gram-negative bacteria) and antifungal properties (targeting *Candida albicans* and non-albicans strains), used in odontostomatology, otorhinolaryngology, and gynecology for its properties. This molecule has a lipophilic nature, showing high affinity with cell membranes and exhibiting membrane stabilization properties, resulting in local anesthesia, an effect related also to the interaction of the drug with cationic channels. In addition, benzydamine HCl is able to inhibit the production of pro-inflammatory cytokines, with consequent analgesia. Moreover, benzydamine HCl is able to inhibit leukocyte-endothelial interactions and platelet aggregation. Unlike other non-steroidal anti-inflammatory drugs, benzydamine HCl does not inhibit cyclooxygenase or lipoxygenase.

 Here we review the most updated clinical data available on benzydamine HCl local application as spray, mouthwash or gargling and evidence of its effectiveness in inflammatory and/or septic conditions in the otorhinolaryngology and odontostomatology settings, with particular reference to sore throat, oral inflammation, dental plaque, tonsillitis/tonsillectomy and chemo- or radiotherapy-induced oral mucositis. Novel formulations for oral administration of benzydamine HCl are also reviewed, including *in situ* gelling formulations to be sprayed onto the damaged oral mucosa. Finally, novel data on the potential role of benzydamine HCl in nociceptor excitability are introduced.

Is the review written in accessible language?

Yes

Are all factual statements correct and adequately supported by citations?

Partly

Are the conclusions drawn appropriate in the context of the current research literature?

Yes

Is the topic of the review discussed comprehensively in the context of the current literature?

Yes

Reviewer Expertise:

Kidney disease and inflammation

I confirm that I have read this submission and believe that I have an appropriate level of expertise to confirm that it is of an acceptable scientific standard, however I have significant reservations, as outlined above.
